# Making everyone count: it is time to improve the visibility of people with disability in primary care

**DOI:** 10.5694/mja2.51650

**Published:** 2022-07-31

**Authors:** Jodie Bailie, Nicola Fortune, Julie Gordon, Richard C Madden, Gwynnyth Llewellyn

**Affiliations:** ^1^ University Centre for Rural Health University of Sydney Lismore NSW; ^2^ Centre for Disability Research and Policy University of Sydney Sydney NSW; ^3^ Centre of Research Excellence in Disability and Health University of Melbourne Melbourne VIC; ^4^ WHO Collaborating Centre for Strengthening Rehabilitation Capacity in Health Systems University of Sydney Sydney NSW

**Keywords:** Primary health care, Registries, Disabled persons

Actions to support identification of people with disability in the Voluntary Patient Registration scheme

Around 4 million people, or 18% of Australia’s population, have a disability.^1^ On average, people with disability experience poorer health outcomes than people without disability as well as inequities in accessing health care.^1^ Primary care — comprising general practice, Aboriginal community‐controlled health services and government‐managed health services — is the frontline of Australia’s health system, and yet there are virtually no data available on access to, or quality of, this care for people with disability.^2^, ^3^, ^4^ Nor is there a standard method to record disability in Australian primary care clinical information systems.^3^, ^5^

Without these data, it is impossible to determine drivers of health inequities, and to develop evidence‐informed policies to improve care and track progress towards reducing health inequities.^2^, ^6^ Submissions to the Royal Commission into Violence, Abuse, Neglect and Exploitation of People with Disability identified lack of data as an impediment to raising awareness about the needs and experiences of people with disability in the health care system, to understanding the issues and barriers, and to making improvements to services.^7^

The purpose of the Voluntary Patient Registration (VPR) scheme, as stated in the Australian Government’s draft Primary Health Care Plan 2022–2032, is to strengthen the continuity of care relationship between individuals and their regular general practice.^8^, ^9^ Under the VPR, Australians can voluntarily register with their preferred general practice by completing a registration form. It is expected that this form will include several population group questions, including one to identify whether the person has a disability and, potentially, a question to determine the person’s disability group.

In this article, we draw on findings from research commissioned by the Australian Department of Health on developing disability questions for the VPR form. Data were collected and thematically analysed from 16 virtual focus groups and four interviews held in late 2021, with input from 65 individuals across 26 organisations throughout Australia. These included organisations supporting people with disability, health care consumer and representative organisations, primary care services, and Primary Health Networks (Box 1).

Box 1 **Number and type of focus group and interview respondent, by organisation and role**

**Table:** 

	Organisations	Participants
Total number of focus groups	26	65
Organisation type		
Disabled people’s organisation/disability representative organisation	9	20
Health consumer representative organisation	2	13
Primary care (eg, general practice)	4	5
Primary Health Network	7	20
Health care representative organisation (eg, RACGP)	4	7
Role type		
General practitioner		10
Medical specialist		3
Nurse/midwife		2
Policy role		2
Project officer/coordinator		17
Senior manager/executive officer		1
Consumer/advocate		30

RACGP = Royal Australian College of General Practitioners. We have reported on the role type identified by respondents. Many respondents held dual roles, for example, advocate and clinician. Two focus groups were conducted to obtain input from people with intellectual disability. Focus groups had a one‐hour duration, except for the two groups seeking input from people with intellectual disability. Focus groups were co‐facilitated by two members of the research team including one team member with lived experience of disability.

Overall, there was broad support for capturing patient disability information on the VPR form as long as this led to improved access to quality primary care and better health outcomes for people with disability. Knowing about patient disability was thought likely to assist general practitioners in being able to provide appropriate health care and put reasonable accessibility adjustments in place. In Box 2 we identify actions needed at multiple levels of the health system for these potential benefits to be realised.

Patient enrolment has been used previously in Australia in Indigenous health care;^10^ it is useful, therefore, to consider learnings from evaluations of the Practice Incentives Program – Indigenous Health Incentive (PIP‐IHI).^10^ Key transferrable lessons are:

- any associated funding model should be structured such that most of the funding is given after a threshold level of care has been provided, rather than payment simply for registration;
- there must be investment in upgrading the existing clinical information system so that practices can effectively identify relevant patients within this system, rather than establishing a parallel system; and
- health professionals will be motivated to participate when this participation offers clinical benefits for patients rather than just being an administrative exercise.

As with the PIP‐IHI enrolment scheme, VPR will include a process for obtaining consent. Ensuring informed consent requires providing information about the nature and purpose of the scheme in a way that patients can understand, establishing systems for people who may need support with consent, and providing guidance around proxy consent.

Evaluation should be undertaken during the first year of the VPR roll‐out to inform ongoing refinements to implementation, such as:

- assessing the data produced by the disability questions relative to specific purposes for which the data are to be used;
- improving the patient experience of completing the VPR form; and
- ensuring that general practitioners’ use of the data about disability benefits patients.

People with disability and their representative organisations must be actively involved in the design and implementation of this evaluation.

The inclusion of disability questions on the VPR form is a step forward in gathering data to highlight gaps in care for people with disability, and to drive policies that will realise their right to the highest attainable standard of health without discrimination, as enshrined in the United Nations Convention on the Rights of Persons with Disabilities.^11^ Attending to the actions outlined in Box 2, and the implementation of a rigorous formative evaluation, will optimise these potential benefits.

Box 2 **Actions to realise the potential benefits of capturing patient disability information as part of the Voluntary Patient Registration (VPR) scheme**

**Table:** 

	Actions
Patient level	The VPR form must be written in a variety of communication formats, including in Easy English and in several community languages, and be available online to ensure that all Australians with disability are able to complete it independently or with their preferred support person. Patients should also have the option of completing the VPR form in consultation with a general practitioner, practice nurse or other health professional, if this is their choice.
Interpersonal level	Provide disability‐related training to clinical and non‐clinical practice staff (eg, receptionists) so as to remove barriers that prevent equitable access to health care by patients with disability. Ensure both clinical and non‐clinical practice staff recognise the value of knowing their patients’ disability status and implications of this for care delivery.
Health service level	The VPR form must be administered respectfully in a private setting, not in the waiting room or reception area, and if assistance is required, this must be sought from a person preferred by the patient. Administrative burden for practices must be minimised, particularly for practices with limited internal capacity for administration. Financial support to improve practice accessibility and quality of care should be provided to practices so they can respond appropriately when patients register their disability information.
Community level	Produce and disseminate Easy English information about the purpose of VPR through multiple channels, including its potential benefits for patients, the protections in place to safeguard the privacy and confidentiality of patient data, and how the data will be used at practice level and by government. This is essential to build trust among all stakeholders.
Policy level	Data captured by the VPR form will need to be integrated into existing clinical information systems to be useful at the practice level, for example, to identify who is registered and to enable development of recall and reminder lists. VPR disability data in clinical information systems would provide opportunities for local quality improvement initiatives to identify gaps and develop action plans for improving quality of care for people with disability. These data could be aggregated to regional and national levels for benchmarking to inform policy and planning. Provision of specific funding arrangements to enable general practitioners to see patients with disability for longer consultations when required.

## Open access

Open access publishing facilitated by The University of Sydney, as part of the Wiley ‐ The University of Sydney agreement via the Council of Australian University Librarians.

## Competing interests

No relevant disclosures.

## Provenance

Not commissioned; externally peer reviewed.
